# Thin Layer Chromatography-Bioautography and Gas Chromatography-Mass Spectrometry of Antimicrobial Leaf Extracts from Philippine *Piper betle* L. against Multidrug-Resistant Bacteria

**DOI:** 10.1155/2016/4976791

**Published:** 2016-07-10

**Authors:** Demetrio L. Valle, Juliana Janet M. Puzon, Esperanza C. Cabrera, Windell L. Rivera

**Affiliations:** ^1^Institute of Biology, College of Science, University of the Philippines, Diliman, 1101 Quezon City, Philippines; ^2^Natural Sciences Research Institute, University of the Philippines, Diliman, 1101 Quezon City, Philippines; ^3^Biology Department, De La Salle University, Taft Avenue, 1004 Manila, Philippines

## Abstract

This study isolated and identified the antimicrobial compounds of Philippine* Piper betle* L. leaf ethanol extracts by thin layer chromatography- (TLC-) bioautography and gas chromatography-mass spectrometry (GC-MS). Initially, TLC separation of the leaf ethanol extracts provided a maximum of eight compounds with *R*_*f*_ values of 0.92, 0.86, 0.76, 0.53, 0.40, 0.25, 0.13, and 0.013, best visualized when inspected under UV 366 nm. Agar-overlay bioautography of the isolated compounds demonstrated two spots with *R*_*f*_ values of 0.86 and 0.13 showing inhibitory activities against two Gram-positive multidrug-resistant (MDR) bacteria, namely, methicillin-resistant* Staphylococcus aureus* and vancomycin-resistant* Enterococcus*. The compound with an *R*_*f*_ value of 0.86 also possessed inhibitory activity against Gram-negative MDR bacteria, namely, carbapenem-resistant Enterobacteriaceae-*Klebsiella pneumoniae* and metallo-*β*-lactamase-producing* Acinetobacter baumannii*. GC-MS was performed to identify the semivolatile and volatile compounds present in the leaf ethanol extracts. Six compounds were identified, four of which are new compounds that have not been mentioned in the medical literature. The chemical compounds isolated include ethyl diazoacetate, tris(trifluoromethyl)phosphine, heptafluorobutyrate, 3-fluoro-2-propynenitrite, 4-(2-propenyl)phenol, and eugenol. The results of this study could lead to the development of novel therapeutic agents capable of dealing with specific diseases that either have weakened reaction or are currently not responsive to existing drugs.

## 1. Introduction

*Piper betle* L. belonging to the family Piperaceae is a climbing vine used in alternative medicine due to its numerous therapeutic properties, which include its antibacterial, antifungal, antioxidant, cytotoxic, antihelminthic, antiprotozoal, antidiabetic, hepatoprotective, and immunomodulatory properties [1]. The plant is known to be widely distributed in India, Sri Lanka, Malaysia, Indonesia, Thailand, China, Philippines, and other subtropical countries [2]. It has been reported to have broad spectrum antimicrobial activities against various bacterial strains [1] and fungi [3, 4].

Results of our previous studies have proven the great potential of* P. betle* as a cure for multidrug-resistant (MDR) bacteria [5, 6]. Out of 12 Philippine medicinal plants subjected to antimicrobial assays, the* P. betle* exceptionally presented significant inhibitory effects against selected MDR isolates [5]. Moreover, the antimicrobial activities of the ethanol, methanol, and supercritical CO_2_ extracts from* P. betle* were determined on clinical isolates of MDR bacteria which have been identified by the Infectious Disease Society of America as being among the currently more challenging strains in clinical management. The assay methods used in the study included the standard disc diffusion method and the broth microdilution method for the determination of the minimum inhibitory concentration (MIC) and the minimum bactericidal concentrations (MBC) of the extracts for the test microorganisms. The study revealed the bactericidal activities of all the* P. betle* leaf crude extracts with MBC ranging from 19 *μ*g/mL to 1250 *μ*g/mL. The extracts proved to be more potent against the Gram-positive methicillin-resistant* Staphylococcus aureus* (MRSA) and vancomycin-resistant* Enterococcus* (VRE) than for the Gram-negative test bacteria. VRE isolates were more susceptible to all the extracts than the MRSA isolates. Generally, the ethanol extracts proved to be more potent than the methanol extracts and supercritical CO_2_ extracts as shown by their lower MICs for both the Gram-positive and Gram-negative MDRs. It is in this light that an investigation on the efficacy of* P. betle* as a source of antimicrobial agents was conducted by performing bioassay-guided isolation and identification of its antimicrobial compounds.

Although there are several studies focusing on the phytochemistry of* P. betle*, there are varying outcomes concerning the constituents or bioactive compounds of the plant, implying that the habitat plays a significant role in the phytochemistry of medicinal plants. One such documentation is a local study made on the Philippine variety of* P. betle*, where it was observed that unlike the Indian variety the major constituents of the essential oil and ether soluble fraction of the Philippine* P. betle* are chavibetol and chavibetol acetate, whereas allylpyrocatechol is the major constituent of the leaves [7]. Information on the particular pharmacologically active constituents of medicinal plants and the subsequent isolation of important compounds are vital in the search for new therapeutic drugs. In the development of new treatment for MDR bacteria, we should be able to identify which bioactive compounds are responsible for significant antagonistic effects against such pathogens, taking into consideration the mechanisms of action and possible toxicity.

Several spectroscopic and chromatographic analytical methods have been developed for standardization of products from medicinal plants, which include mass spectrometry (MS), gas chromatography-mass spectrometry (GC-MS), liquid chromatography (LC), thin layer chromatography (TLC), high performance liquid chromatography (HPLC), and high performance thin layer chromatography (HPTLC) to guarantee their quality, efficacy, and safety [8].

To determine the biologically active natural products in plant extracts, the choice of the bioassay method is critical. The bioassay tests should be sensitive, reliable, simple, and prompt [9]. The type of extraction method, duration of extraction, temperature, and the polarity of solvent used influence the quality and the concentration of bioactive components isolated from the raw material [2, 10]. Activity-guided fractionation is essential, as all fractions should be thoroughly examined and monitored, to detect or identify highly active compounds for further isolation and purification until active monosubstances are obtained [9].

This study aimed to identify and isolate the antibacterial compounds of* P. betle* extracts using TLC, agar-overlay, and contact (indirect) bioautographic assays and to identify the semivolatile and volatile compounds using GC-MS.

## 2. Materials and Methods

### 2.1. Collection and Preparation of Plant Materials

The leaves of the* P. betle* were collected at the foot of Sierra Madre Mountain Range in the Municipality of General Nakar, Quezon, Philippines. The plant was identified and authenticated at the Jose Vera Santos Memorial Herbarium of the Institute of Biology, University of the Philippines, Diliman, Quezon City, Philippines. The leaves were washed thoroughly and then air-dried at room temperature for seven days, finely powdered, and stored in sterile airtight containers until further use.

### 2.2. Preparation of Ethanol Leaf Extracts

Powdered dried leaves of* P. betle* were extracted in accordance with the method of Basri and Fan [11] with minor modifications. Briefly, 150 g of powdered plant material was soaked in 500 mL of ethanol for seven days with occasional stirring and then filtered using Whatman filter paper number 1 (Whatman Ltd., England). The filtrate was concentrated under reduced pressure using a rotary evaporator at 50°C. The crude ethanol extract was collected and allowed to dry at room temperature. The stock solution was prepared by dissolving the dried extract in DMSO at 1 × 10^5^ *µ*g/mL concentration.

### 2.3. Multidrug-Resistant (MDR) Bacterial Strains

The MDR bacterial strains used in this study were the following: Gram-positive MDR bacteria, namely, methicillin-resistant* Staphylococcus aureus* (MRSA) and vancomycin-resistant* Enterococcus* (VRE); Gram-negative MDR bacteria, namely, carbapenem-resistant Enterobacteriaceae- (CRE-)* Klebsiella pneumoniae* and metallo-*β*-lactamase- (M*β*L-) producing* Acinetobacter baumannii*. These MDR bacterial strains were isolated from anonymized patients admitted to Makati Medical Center and Ospital ng Makati. Both are Level III training hospitals located in Makati City, Philippines. All isolates were identified by automated biochemical tests using Vitek®MS (bioMérieux, Marcy l'Etoile, France) GP colorimetric identification card. The susceptibility patterns were obtained by Vitek MS AST (bioMérieux, Marcy l'Etoile, France) following MIC interpretive standard from Clinical Laboratory Standard Institute M100-S24 [12].

### 2.4. Thin Layer Chromatography (TLC)

A TLC system (CAMAG) equipped with a sample applicator was used for application of samples. Five microliters of leaf ethanol extracts was separately applied on 5 cm × 10 cm chromatographic precoated silica gel plates (Merck, TLC grade) as the stationary phase. The TLC plates were developed in a twin trough glass chamber containing mixture of ethyl acetate and* n*-hexane (7 : 3 v/v) as the mobile phase. The plates were removed when the solvent front has moved to 15 cm from the original extract position and subsequently allowed to dry. After drying, the spots on the developed plates were visualized under visible (white), short UV (254 nm), and long UV (366 nm) light. As postderivatization, the plates were sprayed with vanillin-sulfuric acid for color reaction and allowed to dry. A visualizer and a scanner were used for photodocumentation at UV 254 nm and UV 366 nm and under the visible light before and after application of the vanillin-sulfuric acid spray. The movement of each separating spot of the extract was expressed by its retention factor (*R*_*f*_). Values were calculated for each spot using the following formula:

(1) $$\begin{array}{c} R_{f}=\frac{\text{distance traveled by the solute from the point of application to the center of spot}}{\text{distance traveled by the solvent front}}. \end{array}$$

### 2.5. TLC-Contact (Indirect) Bioautography Technique

The inocula of representative MDR bacterial strains with 1.5 × 10^8^ CFU/mL concentration, namely, MRSA, VRE, M*β*L-*A. baumannii*, and CRE-*K. pneumoniae*, were swabbed onto Mueller-Hinton agar plates for use in contact bioautography technique adopted from the method of Wedge and Nagle [13] with slight modifications. The dried TLC plates with corresponding spots were placed aseptically onto the seeded Mueller-Hinton agar plate overlaid with sterile lens paper. The TLC plate was placed face downward with the silica-coated side in contact evenly with the lens paper and was incubated for 12 to 18 hours at 4 ± 2°C. Then, the TLC plate was removed, and the inoculated agar plate was further incubated at 35 ± 2°C for 24 hours in an ambient air incubator. The zone of inhibition was observed and compared with TLC plate *R*_*f*_ value results.

### 2.6. TLC-Agar-Overlay Bioautography Technique

One milliliter of representative MDR bacterial strains with 1.5 × 10^8^ CFU/mL concentration was used for every 10 mL of Mueller-Hinton agar. Developed TLC plates were placed in a sterile Petri dish (150 mm). The culture was added to the melted Mueller-Hinton agar and a thin layer was poured over the TLC plate. After the solidification of the medium, TLC plate was incubated for 24 hours at 35 ± 2°C. The TLC-bioautography plates were sprayed with an aqueous solution (2.5 mg/mL) of methylthiazol tetrazolium (Sigma, USA). Clear zone of inhibition was observed against a purple background [14]. Identification of specific compounds was limited by the unavailability of prepared reference standards.

### 2.7. Gas Chromatography-Mass Spectrometry (GC-MS)

The GC-MS analysis of leaf ethanol extracts was performed using a Perkin Elmer GC-MS (Perkin Elmer Clarus 680 GC-Clarus SQ 8T MS) equipped with Elite-5 MS 30 m × 0.25 mm × 25 *µ*m capillary column (5% diphenyl, 95% dimethylpolysiloxane). For GC-MS detection, an electron ionization system with ionization energy of 70 eV was used. Ultrapure helium gas was used as a carrier gas at a constant flow rate of 1 mL/min. Ion source, mass transfer line, and injector temperature were set at 230°C, 250°C, and 290°C, respectively. The oven temperature was programmed from 50 to 150°C at a rate of 3°C/min and then held in isothermal condition for 10 min and finally raised to 250°C at 10°C/min. Diluted samples (1/100, v/v in ethanol) of 1 *µ*L were manually injected in the split mode of 120. Mass spectral scan range was at 45–450* m/z*, with a solvent delay of 2 min. The components of the extracts were identified based on the comparison of their GC relative retention time and mass spectra with those of NIST MS Search Library Software version 2.0.

## 3. Results and Discussion

### 3.1. Thin Layer Chromatography (TLC)

**Figure 1:**
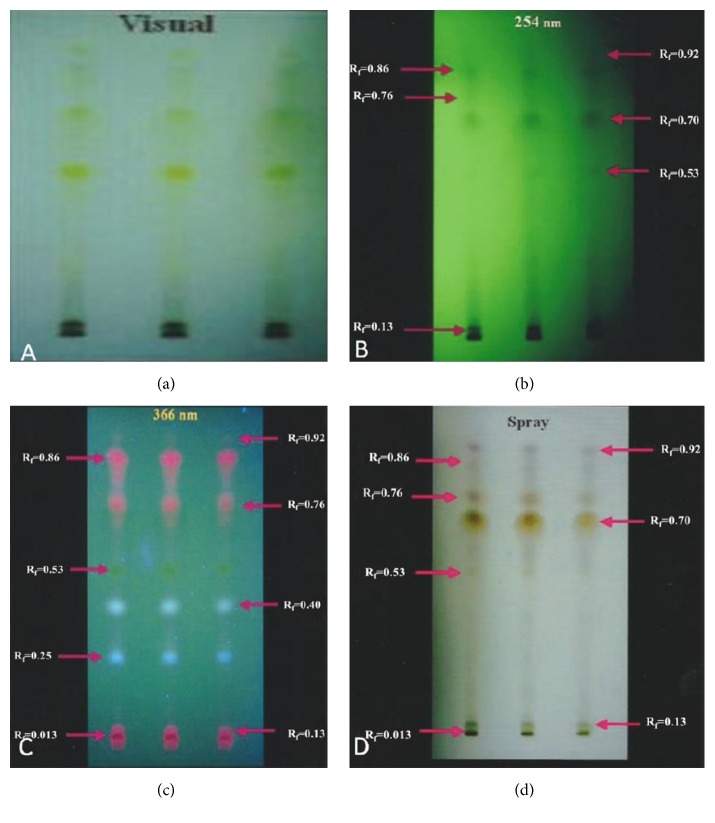
TLC plates of* P. betle* L. leaf ethanol extracts visualized under visible light, UV at 254 nm, and UV at 366 nm and after derivatization with vanillin-sulfuric acid. Mobile phase: ethyl acetate :* n*-hexane (7 : 3 v/v).

To isolate and identify the bioactive compounds of the* P. betle* leaf extract, TLC was initially performed as a qualitative method to document the extract constituents. This method has been widely used to separate secondary metabolites like polyphenols, flavonoids, saponins, alkaloids, and steroids, including amino acids, proteins, peptides, hormones, and pesticides [15]. Although TLC does not provide specific measurements, it is very effective when used in combination with other techniques. In our study, TLC of the* P. betle* leaf ethanol extracts revealed a maximum of 9 compounds in the order of decreasing *R*_*f*_ values, as shown in Table 1. Five bands were evident in the TLC plate visualized under visible light (Figure 1(a)), six bands when viewed under short UV radiation (254 nm wavelength) as shown in Figure 1(b), eight bands when viewed under long UV radiation (366 nm wavelength) as shown in Figure 1(c), and seven bands from the TLC plate sprayed with vanillin-sulfuric acid reagent (Figure 1(d)). The best resolutions were obtained when examined under UV light and after derivatization with the vanillin-sulfuric acid spray. Compounds with *R*_*f*_ values of 0.92, 0.86, and 0.13 were visualized in all TLC chromatograms. It is interesting to note that the compound with *R*_*f*_ 0.70 was evident in all visualization methods, except when viewed under long UV radiation.

### 3.2. TLC-Bioautography

In the agar-overlay and contact (indirect) bioautography assays, the antibacterial activity of the compounds separated on TLC was determined.

**Figure 2:**
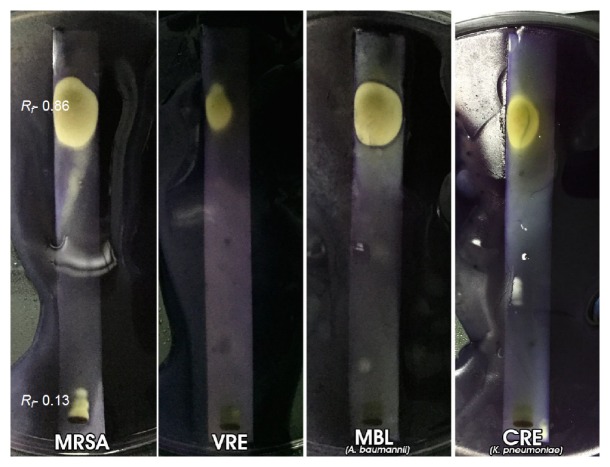
TLC-agar-overlay bioautography of* P. betle* L. leaf ethanol extracts against MRSA, VRE, M*β*L-*A. baumannii*, and CRE-*K. pneumoniae*.

**Figure 3:**
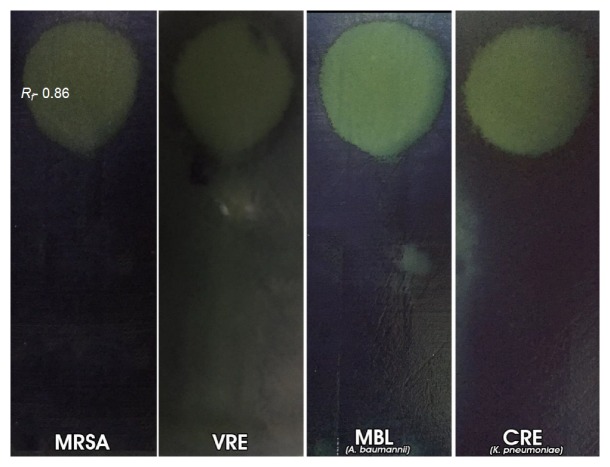
TLC-contact (indirect) bioautography of* P. betle* L. leaf ethanol extracts against MRSA, VRE, M*β*L-*A. baumannii*, and CRE-*K. pneumoniae*.

Representative MDR bacteria from each group with correspondingly similar MIC and MBC values as indicated in parentheses, namely, MRSA (78 *μ*g/mL), VRE (19 *μ*g/mL), M*β*L-*A. baumannii,* (312 *μ*g/mL), and CRE-*K. pneumonia* (312 *μ*g/mL), were used for bioautography. Significant antibacterial activity against MRSA was demonstrated by compounds with *R*_*f*_ values of 0.86 and 0.13 on agar-overlay bioautography, as evident by the significant clear zone of inhibition on a purple background (Figure 2). In the contact (indirect) method, only the compound with *R*_*f*_ 0.86 was visible, shown as a large clear zone of inhibition on a purple background sprayed with methylthiazol tetrazolium (Figure 3). The same compounds were also demonstrated to be active against VRE, as seen on the agar-overlay bioautography result in Figure 2. For the Gram-negative antimicrobial activity, only the compound with *R*_*f*_ value of 0.86 exhibited significant activity against M*β*L-*A. baumannii* and CRE-*K. pneumoniae* (Figures 2 and 3). From the results, a compound with *R*_*f*_ value of 0.86 was found to have inhibitory activity against both Gram-positive and Gram-negative MDR bacteria. Based on earlier studies about TLC of plant compounds, *R*_*f*_ values of 0.86 were exhibited by flavonoids and terpenoids, whereas *R*_*f*_ values of 0.13 were exhibited by alkaloids, saponins, and some flavonoids [16, 17].

The agar-overlay bioautography assay proved to be an excellent method of identifying and localizing compounds with specific antibacterial activity in* P. betle* leaf extract, justifying its value in the search for new antimicrobial agents. It is appropriate for evaluating compounds that can be separated by TLC, against bacteria that grow directly on the TLC plate. This particular assay has the advantages of being cost-effective, rapid, uncomplicated, requiring a simple set-up, a small amount of test sample, and simple interpretation of results, and associated with a high sample output.

### 3.3. GC-MS Analysis

Six volatile and semivolatile compounds in the* P. betle* leaf ethanol extracts were identified based on the comparison of their GC relative retention time and mass spectra with those of NIST MS Search Library Software version 2.0. The antimicrobial compounds identified include ethyl diazoacetate, 4-(2-propenyl)phenol, eugenol, tris(trifluoromethyl)phosphine, heptafluorobutyrate, and 3-fluoro-2-propynenitrite (Table 2). In a previous study on* P. betle* collected from various provinces in the Philippines, namely, La Union, Abra, Iloilo, Palawan, and Bukidnon, twenty compounds were identified as constituents of the plant leaf essential oil [4]. Eugenol is one of the compounds with the highest retention time, as similarly revealed in the result of the GC-MS analysis in our study. Another study of six Indian cultivars of* P. betle* revealed that out of several compounds identified in the leaf extracts eugenol likewise was found to be a common and major compound [18]. Eugenol has documented antibacterial activities against various plant and human pathogens, including an MRSA [19]. Its mechanism of action is reported to be the deformation of macromolecules in the cytoplasmic membrane as verified by FT-IR spectroscopy [20]. Therefore, the presence of high concentration of eugenol and eugenol isomers in the leaf extracts is a possible phytochemical characteristic feature among* P. betle* cultivars, pointing to the potential of the plant species and its cultivars as promising sources of these antimicrobial metabolites against human pathogens. The variation in the chemical composition of the* P. betle* extracts provides evidence that ecological conditions for growth greatly affect the bioactive properties and functions of the medicinal plant.

## 4. Conclusion

The results of this study confirmed the presence of various bioactive compounds in the Philippine variant of* P. betle*, responsible for its different physiological or therapeutic activities. We have identified two compounds via TLC-bioautography with *R*_*f*_ values 0.86 and 0.13 that showed significant activities against selected MDR bacteria. This result points to the potential development of novel therapeutic antimicrobial agents from* P. betle* capable of dealing with specific diseases or medical conditions that either have weakened reaction or are currently not responsive to existing drugs. Analysis through GC-MS has identified the volatile and semivolatile compounds present in the leaf ethanol extract. In-depth phytochemical analysis of the secondary metabolites in* P. betle*, that is, flavonoids, phenolic acids, alkaloids, terpenoids, and saponin, is underway. Future studies are directed towards the development of purified bioactive compounds and quantitative determination of safe concentrations that can be used to improve existing drugs or to create new agents against MDR bacteria.

## Figures and Tables

**Table 1:** Thin layer chromatography (TLC) profile of *P. betle* L. leaf ethanol extract.

*R*_*f*_ value	Visual	UV-254 nm	UV-366 nm	Vanillin-sulfuric acid spray
0.92	Light yellow	Faint dark	Faint red	Violet spot
0.86	Dark gray	Dark gray	Pinkish red	Light brown
0.76	—	Faint dark	Red	Brown
0.70	Green	Dark gray	—	Green
0.53	Yellow	Light dark	Green	Light green
0.40	—	—	Light blue	—
0.25	—	—	Blue	—
0.13	Dark green	Dark gray	Pink	Green
0.013	—	—	Red	Light green

**Table 2:** GC-MS data of *P. betle* L. leaf ethanol extracts.

RT (min)	Compound name	Chemical formula
2.04	Heptafluorobutyrate	C_4_F_7_NaO_2_
2.72	Ethyl diazoacetate	C_4_H_6_N_2_O_2_
5.69	4-(2-Propenyl)phenol	C_9_H_10_O
12.97	3-Fluoro-2-propynenitrite	C_3_FN
8.15	Eugenol	C_10_H_12_O_2_
35.21	Tris(trifluoromethyl)phosphine	C_3_F_9_P
